# Absence of Uptake and Prion-Like Spreading of Alpha-Synuclein and Tau After Intravitreal Injection of Preformed Fibrils

**DOI:** 10.3389/fnagi.2020.614587

**Published:** 2021-01-15

**Authors:** Lien Veys, Jessie Van houcke, Jeroen Aerts, Sophie Van Pottelberge, Michel Mahieu, Audrey Coens, Ronald Melki, Dieder Moechars, Louis De Muynck, Lies De Groef

**Affiliations:** ^1^Laboratory Neural Circuit Development and Regeneration, Department of Biology, KU Leuven, Leuven, Belgium; ^2^Department of Neuroscience, Janssen Research and Development, Division of Janssen Pharmaceutica NV, Beerse, Belgium; ^3^Laboratory of Neurodegenerative Disease, Institute François Jacob, MIRCen, CEA-CNRS, Fontenay aux Roses, France

**Keywords:** alpha-synuclein, tau, intravitreal injection, prion-like spreading, visual system

## Abstract

Although very different in etiology and symptoms, numerous neurodegenerative diseases can be classified as proteinopathies. More so, evidence indicates that the key misfolded proteins at the basis of different neuropathies might share common mechanisms of propagation. As such, the prion-like spreading of protein aggregates through the neural network is subject of intensive research focus and requires adequate models. Here, we made use of the well-defined architecture and large accessibility of the visual system, of which the retinotopic connections represent a simple route of anterograde signaling and an elegant model to investigate transsynaptic, prion-like spreading. In two independent studies, uptake and seeding of alpha-synuclein and tau were examined after intravitreal injection of preformed fibrils. However, extracellular matrix components in the vitreous space and at the vitreoretinal surface appeared to act as a barrier for the entry of both fibrils into the retina. These results show that further experimental refinement is needed to fully realize the potential of the visual system as a model for studying the molecular and cellular mechanisms of anterograde, transsynaptic spreading of prion-like proteins.

## Introduction

A hallmark of many age-related neurodegenerative diseases is the development of abnormal protein aggregates, which follow a spatiotemporal propagation topography throughout the central nervous system (CNS) (Thal et al., 2002; Braak et al., 2004; Jucker and Walker, 2013; Goedert et al., 2014). Indeed, several key proteins have been identified that have prion-like properties and can self-assemble into fibrils, recruit native proteins to transform into proteopathic species and as such seed through the neural network (Brettschneider et al., 2015), e.g., superoxide dismutase 1 (SOD1) and transactive response DNA-binding protein 43 (TDP-43) involved in amyotrophic lateral sclerosis and frontotemporal lobar degeneration (Polymenidou and Cleveland, 2011; Grad and Cashman, 2014; Smethurst et al., 2015; Ayers et al., 2016), tau and amyloid-beta (Aβ) underlying Alzheimer’s disease (AD) (Walker et al., 2016; Ayers et al., 2018) and alpha-synuclein (α-syn) at the basis of Parkinson’s disease (PD) (Luk et al., 2012; Goedert, 2015; Herva and Spillantini, 2015; Peelaerts et al., 2015; Ma et al., 2019). Yet, despite evidence pointing toward common routes of propagation, which provides an opportunity to halt or treat numerous proteinopathies, the exact molecular mechanisms of prion-like transmission are still poorly understood.

The visual pathway has since long emerged as an excellent model system to gain insights into classical neurodegenerative diseases, as not only the brain but also the retina is often affected in proteinopathies (Armstrong, 2009; Martínez-Lapiscina et al., 2014; Rahimi et al., 2015; Veys et al., 2019). In Creutzfeldt-Jacob disease (Head et al., 2005; Orrù et al., 2018), as well as PD (Ortuno-Lizaran et al., 2018; Veys et al., 2019) and AD (Hart et al., 2016), patients have been shown to display abnormal retinal accumulation of prion protein-scrapie (PrP^*Sc*^), α-syn, tau, and/or Aβ, respectively. In addition, the spreading of prion pathology to the retina following PrP^*Sc*^ brain inoculation (West Greenlee et al., 2016) as well as the anterograde transmission of PrP^*Sc*^ species from eye to brain (Fraser, 1982; Fraser and Dickinson, 1985; Kimberlin and Walker, 1986; Scott and Fraser, 1989a,b; Foster et al., 1990; Liberski et al., 1990, 2012; Scott et al., 1991, 1992; Jeffrey et al., 1995; Polanco et al., 2017), has been demonstrated in rodents.

The real value of the visual system as a seeding model lies in its well-characterized anatomy and stratified organization, where the optic nerve is the sole anatomical connection to the brain, bundling the axons of only one type of retinofugal projection neurons, i.e., the retinal ganglion cells (London et al., 2013), and thus providing a true model of transsynaptic transmission. In addition, it has the advantage of excluding passive uptake or off target spreading of injected substances to other brain areas, a phenomenon that is occurring when doing intracerebral injections of proteopathic species or human brain homogenates to assess their infectious properties (Clavaguera et al., 2013; Watts et al., 2013; Recasens and Dehay, 2014; Jones et al., 2015; Guo et al., 2016; He et al., 2018; Smolek et al., 2019).

In this study, we aimed to use the eye as a convenient entry route to the CNS, to investigate the uptake and spreading of α-syn and tau preformed fibrils (PFFs). However, in contrast to the findings using PrP^*Sc*^ species, we here report that both α-syn and tau PFFs do not enter the retinal parenchyma after intravitreal (IVT) injection and therefore cannot seed aggregation in the retina or visual target brain areas. Our results suggest that the extracellular matrix (ECM) components in the vitreous space and at the vitreoretinal surface [posterior vitreous membrane and/or inner limiting membrane (ILM)] are a strong barrier against retinal uptake of both prion-like proteins and hence severely hamper the possibility of studying anterograde spreading of α-syn and tau along the visual pathway.

## Materials and Methods

### Animals

Wild-type C57Bl/6 were bred under standard laboratory conditions and were 2–4 months old at the start of the experiments. All experiments were performed according to the European directive 2010/63/EU and in compliance with protocols approved by the local ethical committee. The number of animals (n) used in each experiment is indicated in the figure legends.

### Fibril Preparation and Intravitreal Injections

Expression and purification of human wild-type α-syn was performed as previously described (Ghee et al., 2005), followed by α-syn PFF formation according to the protocol of Bieri et al. (2019). α-syn was assembled into fibrillar polymorphs and labeled with Atto 488 NHS-ester (Atto-Tec, AD 488-3) prior to being fragmented by sonication (Shrivastava et al., 2020). The resulting PFFs are cylindrically elongated polymers and have an average size of 50nm after sonication, as described previously by Bieri et al. (2019) and Peelaerts et al. (2015), and exhibit seeding and spreading activity both *in vitro* (Freundt et al., 2012; Bieri et al., 2019) and *in vivo* in mice and rats (Peelaerts et al., 2015; Bieri et al., 2019; Rey et al., 2019). Atto 488-labeled bovine serum albumin (BSA) was used as a control. Truncated human tau fragments (K18; residues Q244-E372 of 2N4R tau) with a P301L mutation were custom produced (Tebu-Bio). Tau fibrillation was achieved as described before (Peeraer et al., 2015) using low molecular weight heparin (Sigma-Aldrich, H-5284), and Tau K18 PFFs were shortly sonicated prior to injection. Such K18 PFFs have proven their *in vitro/in vivo* spreading and seeding capacity and efficacy to induce pathology *in vivo* in the mouse brain (Calafate et al., 2015; Peeraer et al., 2015; Verheyen et al., 2018; Detrez et al., 2019). Either 5 μg α-syn PFFs, 10 μg tau K18 PFFs or controls (5 μg BSA or vehicle PBS) were intravitreally injected as described before (De Groef et al., 2016). For enzymatic digestion, 5 μg α-syn PFFs was co-injected with either 0.05 μg pronase E or a combination of 1U heparinase III and hyaluronan lyase.

### Confocal Scanning Laser Ophthalmoscopy

*In vivo* confocal scanning laser ophthalmoscopy (cSLO) was performed using a Heidelberg Spectralis set-up (Heidelberg Engineering) with a 50° lens and 488 nm excitation light, to image Atto 488-labeled α-syn PFFs in the dilated eye of anesthetized animals.

### Histology and Immunohistochemistry

Mice were humanely euthanized by IP injection of sodium pentobarbital (60 mg/kg, Dolethal, Vetoquinol) followed by transcardial perfusion, or decapitation. The eyes and brains were dissected, fixed in a formalin-based fixative for 4 h at 4°C and embedded in paraffin. Immunohistochemical (IHC) stainings were performed on 5–7 μm sagittal sections. In short, sections were first deparaffinized, and a heat induced antigen retrieval in citrate buffer (pH 6.0) was used. After blocking of endogenous peroxidase activity with 0.3% hydrogen peroxide and blocking with pre-immune serum, the samples were incubated with one of the following primary antibodies: human specific α-syn [0.1 μg/ml; Millipore (clone Syn211) 36-008], total α-syn (0.5 μg/ml; BD Transduction laboratories 610787), phospho-S129 α-syn [6 μg/ml; Abcam (EP1536Y) ab51253], CD45 (5 μg/ml; BD Pharmingen 553076), F4/80 (10 μg/ml; Bio-Rad MCA 497 G), myeloperoxidase (MPO) (5 μg/ml; Dako, A-0398), GFAP (1 μg/ml; Dako #Z0334), Aquaporin 4 (0.1 μg/ml; Alomone Labs 249-323), laminin (5 μg/ml; Sigma L9393), PT76 (0.05 μg/ml; developed in-house). Labeling for fluorescence microscopy was performed using Alexa labeled secondary antibodies (Jackson) or with a tyramid signal amplification protocol (PerkinElmer). Slides were then counterstained with 4′,6-diamidino-2-phenylindole (DAPI) and mounted with mowiol (Sigma-Aldrich) mounting medium. Imaging was performed using an Olympus FV1000 confocal microscope (Olympus Corp.). Labeling for bright field microscopy was performed using peroxidase labeled secondary antibodies (Dako), followed by a 3,3-diaminobenzidine color reaction (Dako). Slides were counterstained with hematoxylin, dehydrated and mounted with Vectamount permanent mounting medium (Vector Labs). Imaging was performed using a virtual slide scanner (NanoZoomer XR, Hamamatsu).

### Image Analysis

The number of CD45-positive cells in the vitreous was quantified, using Fiji software (Schindelin et al., 2012), on five transverse sections per mouse, including the central section containing the optic nerve head, and the sections located 210 and 420 μm anterior/posterior. The total α-syn immunopositive area was measured in the inner retina (from the retinal nerve fiber layer until the bottom of the inner nuclear layer included) over a distance of 300 μm at four locations per section on five transverse sections per mouse. Average numbers of infiltrating cells and immunopositive area per mouse were compared between α-syn PFFs, BSA and PBS injected eyes with a one-way ANOVA, using GraphPad Prism (v8.4.3).

## Results

### Case Study 1: Absence of α-syn PFF Uptake in the Retina After IVT Injection

C57Bl6/J mice received an IVT injection of 5 μg Atto 488-labeled α-syn PFFs, based on previous reports (Bieri et al., 2019; Zhang et al., 2019). In a dose-response study, we tested 1.25, 2.5, 5, and 10 μg of Atto 488-labeled α-syn PFFs and found no adverse effects on visual performance or retinal morphology up till 14 days after bolus injection, as assessed via the optomotor test and using optical coherence tomography, respectively (data not shown).

**FIGURE 1:**
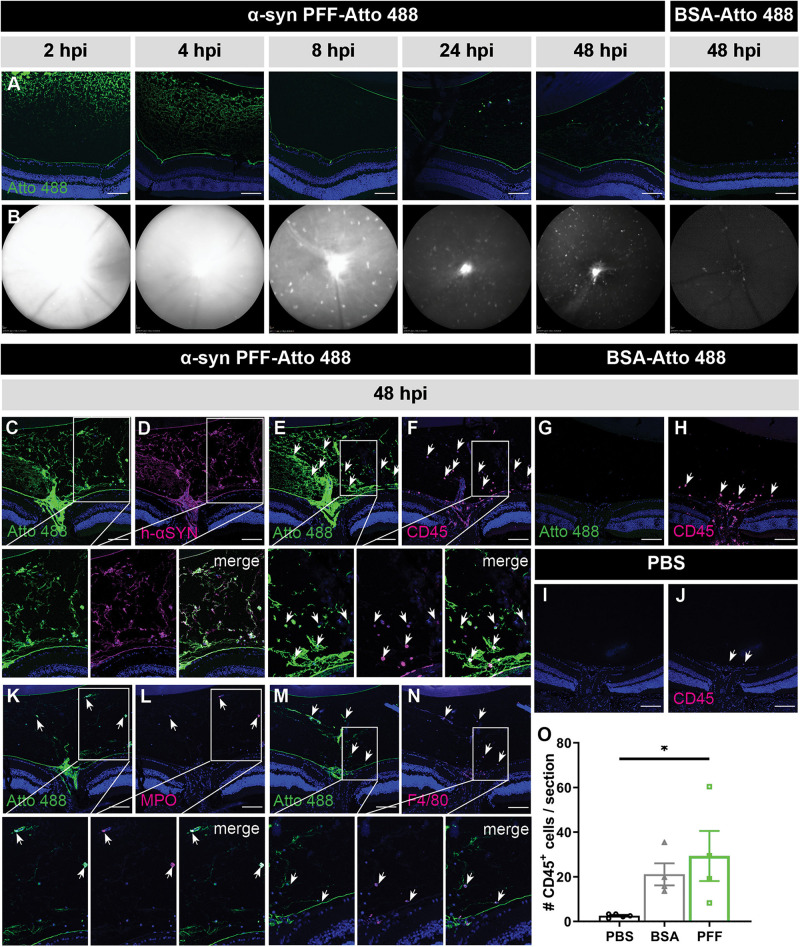
Short term localization of Atto 488-labeled α-syn PFFs or BSA after IVT injection. **(A)** Visualization of the Atto 488 tag on sagittal eye sections demonstrates the localization of α-syn PFFs at 2, 4, 8, 24, and 48 h post IVT injection; and of BSA at 48 h post IVT injection. **(B)** Histology is confirmed by *in vivo* cSLO imaging of the same mice. **(C,D)** Immunostaining for human α-syn demonstrates co-localization of the α-syn PFFs with the Atto 488 tag at 48 h post IVT injection. **(E,F)** Immunostaining of infiltrating immune cells (CD45) on sagittal eye sections shows co-localization with the Atto 488 label (arrows) at 48 h post α-syn PFF IVT injection. **(G,H)** At 48 h post IVT injection of the BSA control, the Atto 488 label is absent in the vitreous yet a limited infiltration of CD45^+^ inflammatory cells is seen (arrows). **(I,J)** At 48 h post IVT injection of PBS (vehicle), infiltration of CD45^+^ inflammatory cells (arrows) is nearly absent. **(K–N)** The identity of the CD45^+^ infiltrating immune cells was further explored via immunostaining for MPO **(K,L)** and F4/80 **(M,N)**. These demonstrate that the Atto 488 tag is present in neutrophils and macrophages, respectively, at 48 h post IVT injection. **(O)** The number of CD45^+^ cells in the vitreous was quantified at 48 h post IVT injection of PBS, BSA or α-syn PFFs. Data are shown as mean ±SEM, ^∗^P < 0.05. Scalebar: 100 μm, *n* = 3–8. hpi, hours post injection.

Localization of Atto 488-labeled α-syn PFFs was examined at 2, 4, 8, 24, and 48 h post IVT injection via IHC on sagittal eye sections (Figure 1A) and by *in vivo* cSLO imaging (Figure 1B). At all time points, in all samples, α-syn PFFs were detected in the vitreous, especially near the lens and ILM, while fluorescent signal was absent in the retina. Immunostaining for human α-syn confirmed this localization (Figures 1C,D). Both IHC and cSLO imaging revealed a strong diffuse Atto 488 signal in the vitreous at 2 and 4 h post injection, which diminished thereafter. By 48 h, most of the diffuse Atto 488-labeled α-syn PFFs had been cleared from the vitreous cavity. Furthermore, from 8 h onward, we observed fluorescent spots on the cSLO images, predominantly around the optic nerve head, but also more dispersed in the rest of the vitreous. Immunostainings on sagittal eye sections identified these as infiltrating immune cells (i.e., neutrophils and monocyte-derived macrophages) that had taken up the PFFs, as shown by co-localization of CD45, MPO, and F4/80 immune cell markers and the Atto 488-label (Figures 1E,F,K–N). Of note, these immune cells were also seen in control mice injected with Atto 488-labeled BSA (Figures 1B,G,H,O), but not in PBS treated animals (Figures 1I,J,O). Together, these data suggest that α-syn PFFs do not enter the retina within the first two days after IVT injection yet remain in the vitreous, where they elicit an immune response and are next engulfed by infiltrating immune cells.

**FIGURE 2:**
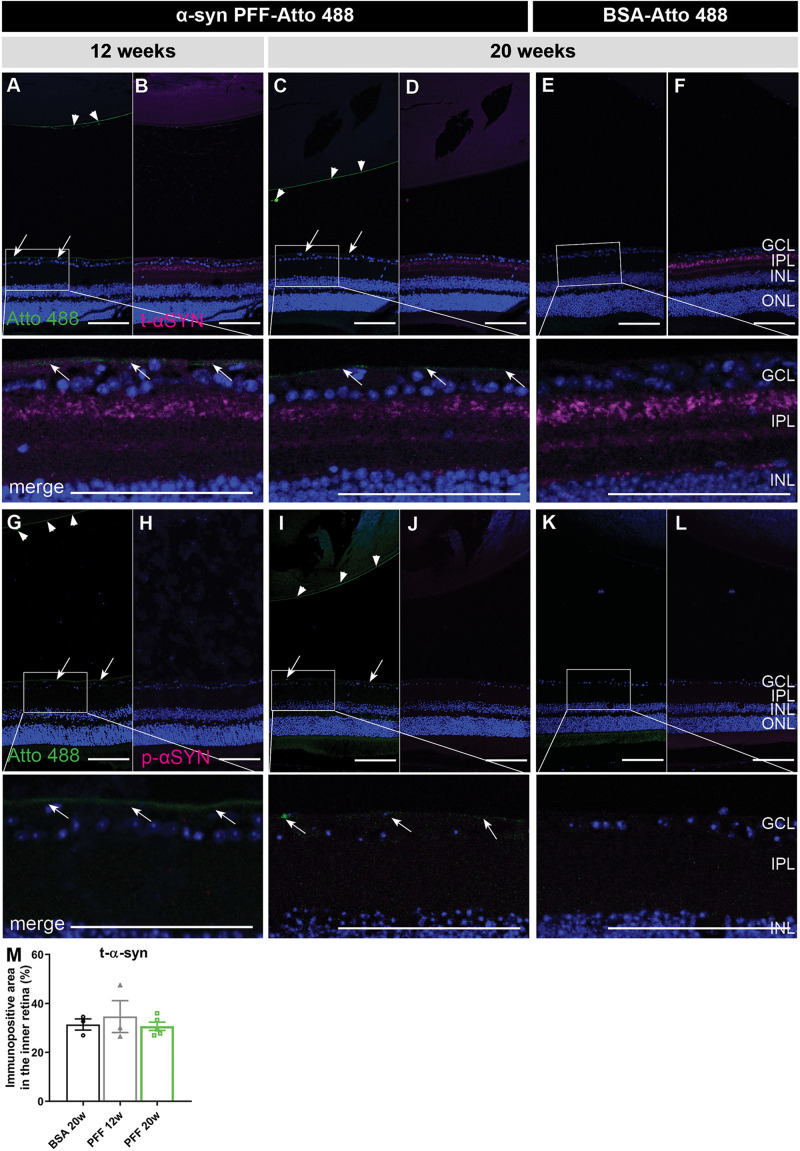
Localization of α-syn in the eye at 12 and 20 weeks post IVT injection of α-syn PFFs. **(A–D)** Histological examination of sagittal eye sections demonstrates the absence of Atto 488-labeled α-syn PFFs in the retina at 12 weeks **(A,B)** and 20 weeks **(C,D)** post IVT injection. Limited Atto 488 signal is detected in the vitreous, predominantly at the ILM (arrows) and lens capsule (arrow heads). **(E,F)** Atto 488-labeled BSA is detected in retina nor vitreous at 20 weeks post injection. **(A–F)** Immunohistochemistry for total α-syn reveals presence of PFFs and endogenous α-syn, distributed similarly in the retina of eyes injected with α-syn PFF-Atto 488 and BSA-Atto 488. **(G–L)** Immunohistochemistry for p-α-syn shows its absence in the retina of eyes injected with α-syn PFF-Atto 488 and BSA-Atto 488. **(M)** Total α-syn in the retina was quantified by measuring the immunopositive area in the inner retina of animals injected with BSA (20 weeks) or α-syn PFFs (12 and 20 weeks post injection). Scalebar: 100 μm, *n* = 4. GCL, ganglion cell layer; IPL, inner plexiform layer; INL, inner nuclear layer; ONL, outer nuclear layer.

Hypothetically, just a small amount of α-syn fibrils – potentially remaining under the detection limit in the experiments above –, could induce a seeding effect (i.e., aggregation of endogenous α-syn induced by exogenous α-syn PFFs) and thereby be sufficient to induce pathology over time (Goedert et al., 2017). Therefore, we assessed the presence of α-syn PFFs, total α-syn (i.e., endogenous and exogenous α-syn combined) and phosphorylated (Ser129) α-syn (p-α-syn, i.e., pathological, seeded α-syn) at 12 and 20 weeks post injection (Figure 2). α-syn PFFs were still present in the vitreous at 12 and 20 weeks, although to a much lesser extent than at the previously investigated early time points, and predominantly located at the ILM and lens capsule (Figures 2A–D,G–J). They were absent in the retina. No traces of Atto 488-labeled BSA were found in both retina and vitreous (Figures 2E,F,K,L). Immunohistochemistry for total α-syn did not reveal a difference in staining pattern nor immunopositive area between PFF-Atto 488- and BSA-Atto 488-injected eyes (Figure 2M). More importantly, no p-α-syn was detected in the retina 12 and 20 weeks after injection of α-syn PFFs (Figures 2G–J) or BSA (Figure 2K,L), hence, no seeding effect was observed with immunohistochemistry.

**FIGURE 3:**
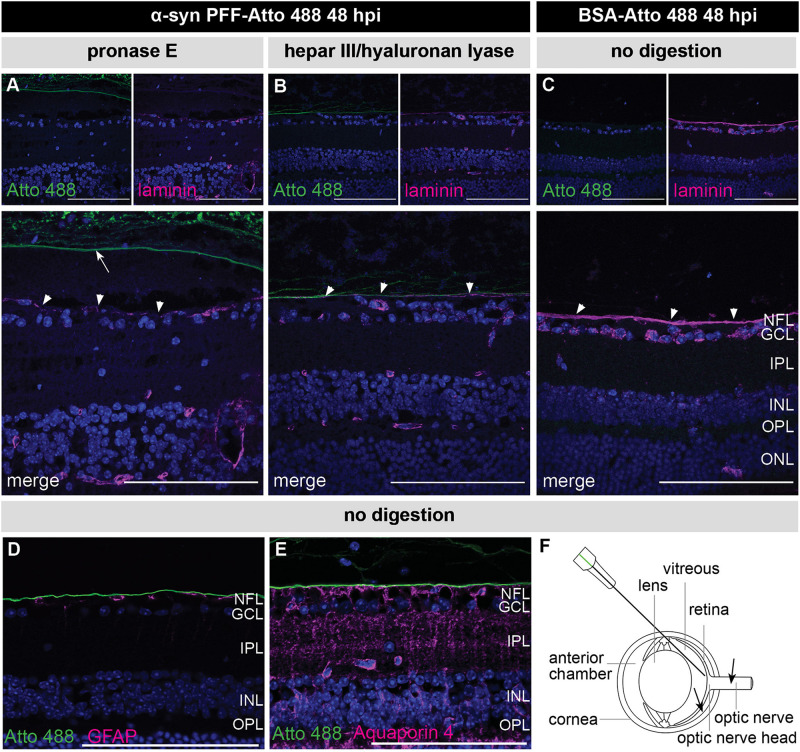
Confirmation of enzymatic digestion of the ILM yet no retinal PFF uptake at 48 h post IVT co-injection of α-syn PFFs and enzymes. **(A,B)** Histological examination of sagittal eye sections demonstrates the absence of Atto 488-labeled α-syn PFFs in the retina after co-injection of α-syn PFFs with **(A)** pronase E or **(B)** a combination of heparinase III and hyaluronan lyase. Immunostaining for laminin, one of the main constituents of the ILM, demonstrates ILM degradation (arrowheads) after enzymatic digestion. Note the posterior vitreous detachment induced by the pronase E treatment (arrow). **(C)** An intact ILM (arrowheads) was seen in the control condition, i.e., 48 h post injection of Atto 488-labeled BSA without enzymatic digestion. **(D,E)** Immunostaining for GFAP (P) and Aquaporin 4 (Q) reveals absence of the Atto 488 tag in astrocytes and Müller glia end feet at 48 h post IVT injection of Atto 488-labeled α-syn PFFs without digestion. **(F)** Schematic drawing of the eye and the IVT injection route. Alternative injection routes (subretinal, in the optic nerve) are indicated with arrows. Scalebar: 100 μm, *n* = 4. hpi, hours post injection; NFL, nerve fiber layer; GCL, ganglion cell layer; IPL, inner plexiform layer; INL, inner nuclear layer; OPL, outer plexiform layer; ONL, outer nuclear layer.

A recurring observation in these experiments is that the α-syn PFFs specifically localize to the ILM, vitreous and lens capsule. These all have in common an ECM composition consisting of collagen and glycosaminoglycans. Given previous reports of α-syn – as well as tau, prion protein (PrP) and Aβ – having a high affinity for sulfated glycosaminoglycans (reviewed in Shrivastava et al., 2017), we hypothesized that the α-syn PFFs may adhere to these structures and are thereby trapped before being able to enter the retina (Parnetti and Cornelli, 2007; Holmes et al., 2013; Puangmalai et al., 2020). A similar phenomenon has been observed for AAV2 vectors: naturally occurring AAV2 serotypes produce limited retinal transduction because the vitreous, ILM, retinal ECM, and cell surface proteoglycans form substantial barriers and binding sites that immobilize the AAV particles (Cehajic-Kapetanovic et al., 2018). Interestingly, retinal AAV2 transduction was increased by co-injection of glycosaminoglycan-degrading enzymes. Adopting the same approach, we co-injected pronase E, a mixture of peptidases hydrolyzing glycoproteins, or a combination of heparinase III and hyaluronan lyase, with the α-syn PFFs (Dalkara et al., 2009; Cehajic-Kapetanovic et al., 2018). Nevertheless, although the effectivity of the enzymatic digestions was confirmed with a laminin staining (Figures 3A–C), as previously described in Dalkara et al. (2009), these treatments did not promote α-syn PFF infiltration in the retina (Figures 3A,B). Of note, besides ECM, the ILM is also composed of astrocytes and Müller glia end feet. Immunostainings for GFAP (astrocytes) and Aquaporin 4 (Müller glia end feet) and α-syn PFFs confirmed that these glial cells are also not taking up any α-syn PFFs (Figures 3D,E).

### Case Study 2: IVT Injection of Tau K18 PFFs Confirms Lack of Retinal Uptake

**FIGURE 4:**
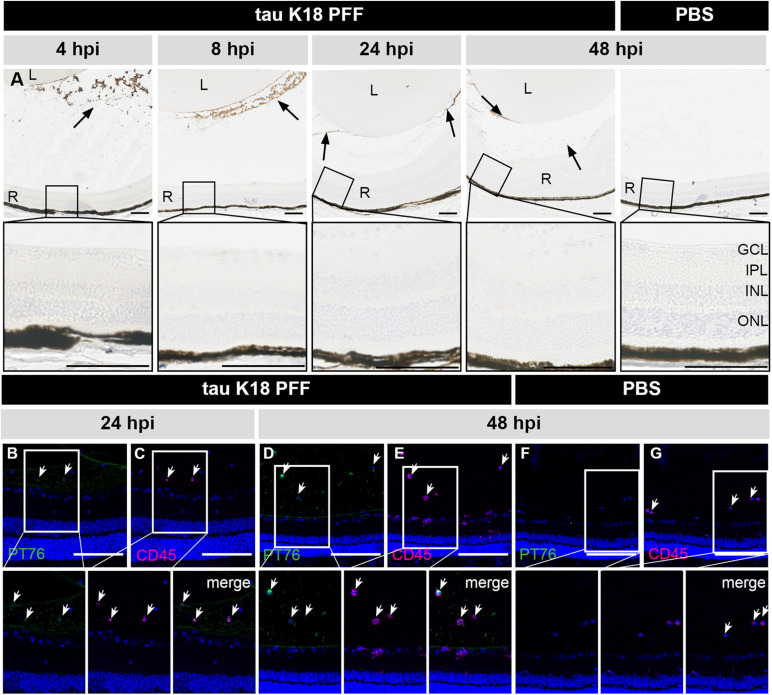
Short term localization of tau K18 PFFs in the eye post IVT injection. **(A)** Histological examination of sagittal eye sections with the PT76 antibody demonstrates the localization of tau K18 PFFs (arrows). Upper row: in the condition where tau K18 PFFs were injected, PT76 staining can be observed in the vitreous body while no staining in the retina is apparent at 2, 4, 8, 24, and 48 h post IVT injection. In the PBS injected condition, no PT76 staining is observed in the vitreous body or the retina at 48 h post IVT injection. Lower row: sagittal sections of the retina, with the retinal layers highlighted, show the lack of any PT76 signal in the tau K18 PFFs conditions. **(B–E)** Immunostaining of infiltrating immune cells (CD45) on sagittal eye sections shows co-localization with PT76 at 24 and 48 h post K18 PFF IVT injection. **(F,G)** At 48 h post IVT injection of the PBS control, PT76 staining is absent in the vitreous and infiltration of CD45^+^ inflammatory cells is limited. Scalebar: 100 μm, *n* = 4 per time point. L, lens; R, retina; GCL, ganglion cell layer; IPL, inner plexiform layer; INL, inner nuclear layer; ONL, outer nuclear layer.

In a second independent study, a similar approach was used to investigate retinal uptake of tau seeds. C57Bl6/J mice were injected IVT with 10 μg tau K18 PFFs (Peeraer et al., 2015), which were visualized at 4, 8, 24, and 48 h post IVT injection using the PT76 antibody [binding specifically to AA 249–256 within the 4R domain of human tau (Vandermeeren et al., 2018)] (Figure 4A). As seen for α-syn PFFs, tau K18 PFFs were detected in the vitreous, at the lens capsule and ILM at all time points studied. No signal was observed in the PBS condition. Despite tau K18 PFFs being present in the eye for up to 2 days, PT76 signal was completely absent from the retina at each of the time points studied, and instead engulfed by inflammatory CD45^+^ cells in the vitreous (Figures 4B–G). Altogether, these data show that IVT injected tau K18 PFFs cannot enter the retina, similar to the results obtained using α-syn PFFs.

## Discussion

Today, many research efforts are focusing on elucidating the (common) underlying mechanisms of protein propagation, as inhibiting the spreading of proteotoxic species through the neural network would block disease progression of numerous neuropathologies; despite the variation in prevalence, location, function, and initial structure of their respective misfolded proteins. Here, we made use of the visual system to study the uptake and seeding mechanisms of α-syn and tau PFFs, two pathological fibril species extensively studied in various *in vitro* and *in vivo* models to investigate their propagation properties (Kirik et al., 2003; Eslamboli et al., 2007; Clavaguera et al., 2009, 2013; Desplats et al., 2009; Hansen et al., 2011; Angot et al., 2012; Breydo et al., 2012; Holmes et al., 2013; Reyes et al., 2014; Calafate et al., 2015; Peelaerts et al., 2015; Stancu et al., 2015; Rey et al., 2016; Wang and Mandelkow, 2016; Shimozawa et al., 2017; Stopschinski and Diamond, 2017; Kundel et al., 2018). However, limitations of these current models, such as the presence of complex anterograde and retrograde connections, prevent straightforward analysis of true transsynaptic propagation. In this regard, the visual system represents an elegant *in vivo* model in which the route of transmission is reduced to a simple anterograde pathway from the retinal ganglion cells, via their axons that form the optic nerve, to the dorsal lateral geniculate nucleus and the superficial layers of the superior colliculus. These well-defined, retinofugal connections allow unbiased assessment of transsynaptic spreading as they provide the additional advantage of excluding aggregation arising from the passive uptake of α-syn and/or tau seeds by neurons. Yet, despite being a promising approach to study protein propagation, our results show no retinal uptake of either α-syn or tau K18 PFFs after IVT injection into the mouse eye.

The lack of α-syn or tau propagation in the visual system upon intravitreal injection is in sharp contrast with previous PrP^*Sc*^ research showing successful infection of the visual system after inoculation in either the anterior eye chamber or vitreous (Fraser, 1982; Fraser and Dickinson, 1985; Kimberlin and Walker, 1986; Scott and Fraser, 1989a,b; Foster et al., 1990; Liberski et al., 1990, 2012; Scott et al., 1991, 1992; Jeffrey et al., 1995; Polanco et al., 2017). This discrepancy in PrP^*Sc*^ versus PFF uptake in the retina and subsequent propagation in the visual system could lie in the differences in the intrinsic nature of prions versus prion-like proteins, such as α-syn and Tau. Indeed, albeit this is not yet fully elucidated, the structural organization of critical domains of prion(-like) proteins appears to determine their infectivity, replication, and propagation. As such, differences in protein aggregate conformation – so-called strains – translate into differences in infectivity, resistance to clearance, host range, and the ability to target specific brain structures. Accordingly, studies of synthetic prions/prion-like proteins have revealed that not all strains are replication and propagation competent and, if they are, induce neuropathological changes characterized by a unique pattern of protein deposition in defined brain regions (Scott and Fraser, 1989a; Foster et al., 1990; Scott et al., 1992; Sanders et al., 2014; Shrivastava et al., 2017; Ghag et al., 2018). As such, while the α-syn and tau strains used in this study have been proven to induce seeding, propagation, and pathology upon brain inoculation, they might not have the optimal structural and/or biochemical properties to do so in the retina. Or, alternatively, the environment of the retina might be less permissive to their prion-like propagation. In this respect, it is important to note that PrP^*Sc*^ is a true prion, with high infectivity (i.e., transmission between individuals) and efficient replication and propagation. In comparison, besides being non-infectious (Ma et al., 2019), tau and α-syn pathology seem to spread more slowly (Brundin et al., 2010; Vasili et al., 2019). These speculations on the difference in seeding potential of PrP^*Sc*^ versus α-syn and tau PFFs, in the brain versus retina, are corroborated by the observation that the α-syn and tau PFFs used in this study have been widely deployed in intracerebral injection paradigms, where using the same dose, pathology was elicited a few weeks after injection (Luk et al., 2012; Peelaerts et al., 2015; Peeraer et al., 2015; Stancu et al., 2015; Bieri et al., 2019). Second, experimental transmission of PrPs is more efficient via intracerebral injections when compared to intraocular, intraspinal, intraperitoneal, and subcutaneous injections (Kimberlin and Walker, 1986; Scott and Fraser, 1989a; Scott et al., 1992). For scrapie, it has been described that compared to a standard intracerebral injection, intraocular injection produces a 30–60% prolongation in incubation period for all mouse models so far examined (Scott et al., 1992). Therefore, α-syn aggregation levels were studied in the retina at 12 and 20 weeks post IVT injection. However, no signs of α-syn pathology were detected in the retina with immunohistochemistry, yet a more sensitive assay should be included in future studies to confirm the lack of seeding in the retina.

As an alternative, not mutually exclusive, explanation for the lack of α-syn and tau propagation observed in this study, we propose that their uptake into the retinal neurons is hampered by intrinsic biological barriers in the eye/retina that prevent entry of IVT injected α-syn and tau K18 PFFs. Results from this study suggest that glycosaminoglycans may be an important constituent of this intrinsic barrier. Indeed, a recurring observation is that the injected PFFs specifically localize to the ILM, vitreous and lens capsule, which have in common an ECM composition with glycosaminoglycans. In this regard, it is known that α-syn, as well as tau, PrP, and Aβ, have a high affinity for sulfated glycosaminoglycans, such as hyaluronic acid, heparan sulfate proteoglycans (HSPGs), and chondroitin sulfate proteoglycans (Parnetti and Cornelli, 2007; Holmes et al., 2013; Puangmalai et al., 2020). Different HSPGs have been described to facilitate the cellular internalization of α-syn and tau *in vitro* and *in vivo*, and blocking their expression diminishes the internalization of tau and α-syn monomers and aggregates (Holmes et al., 2013; Gerson et al., 2014; Dujardin et al., 2018). We propose that α-syn and tau PFFs are trapped by these ECM constituents and are thus prevented from entering the retina. Myeloid cells next clear the PFFs from the vitreous space (Parnetti and Cornelli, 2007; Holmes et al., 2013; Puangmalai et al., 2020). Although intravitreal injection is the preferred injection route to target retinal ganglion cells, for the sake of substantiating this hypothesis, one could perform subretinal injections or direct injection in the optic nerve to surpass these barriers (Figure 3F).

Notably, a similar mechanism of heparan sulfate-mediated uptake has been described for AAV2 vectors, and these vectors also appear to be trapped at the ILM upon IVT injection. Co-injection of glycosidic enzymes is an effective method of increasing AAV2-mediated transduction of the retina and hence we applied this mitigation strategy to our IVT α-syn PFF injections. However, while we confirmed digestion of the ILM, we did not observe any increase in retinal uptake. This suggests that cellular uptake of α-syn and tau PFFs in the retina is dependent either on different receptors or on alternative mechanisms, or restrained by different factors. This highlights our limited understanding of the mechanisms for α-syn and tau cellular uptake in the CNS. Of note, besides ECM, the ILM is composed of astrocytes and Müller glia end feet. Although astrocytes are known to easily internalize α-syn and tau species in the brain, this appears not to be the case in the retina.

Altogether, our results suggest that ECM components in the vitreous space and at the vitreoretinal surface (posterior vitreous membrane and/or ILM) may act as a barrier that prevents the entry of α-syn and tau PFFs into the healthy retina. The exact physicochemical interactions preventing PFF uptake are as yet unidentified, and alternative injection routes (e.g., subretinal injection) or strategies for local resolution of the ECM and/or facilitation of cellular uptake are needed to enhance penetrance of the PFFs into the retinal parenchyma. Furthermore, a comparative study of different α-syn and tau strains might shed light on which conformers have the highest replication and propagation competence in the retina/visual system. Together, these follow-up studies will be essential to the further development of the visual system as an anterograde seeding model and ultimately a deeper understanding of the molecular mechanisms involved in transsynaptic spreading of prion-like proteins.

## Data Availability Statement

The raw data supporting the conclusions of this article will be made available by the authors, without undue reservation.

## Ethics Statement

The animal study was reviewed and approved by the KU Leuven and Janssen Research and Development institutional ethics committees for animal research.

## Author Contributions

LV, DM, LDM, and LDG contributed to the conception of the study. LV, JV, JA, and RM elaborated on the study design. LV, JV, JA, SV, MM, and AC performed the experimental work. LV, JV, and LDG wrote the manuscript. RM, DM, and LDM edited the manuscript. All authors contributed to the article and approved the submitted version.

## Conflict of Interest

The authors declare that the research was conducted in the absence of any commercial or financial relationships that could be construed as a potential conflict of interest.

## Abbreviations

A β: Amyloid-beta
AD: Alzheimer’s disease
α-syn: alpha-synuclein
BSA: bovine serum albumin
CNS: central nervous system
DAPI: 4 ′,6-diamidino-2-phenylindole
ECM: extracellular matrix
HSPG: heparan sulfate proteoglycan
Htt: huntingtin protein
IHC: immunohistochemistry
ILM: inner limiting membrane
IVT: intravitreal
IP: intraperitoneal
MPO: myeloperoxidase
p- α-syn: phosphorylated alpha-synuclein (Ser129)
PD: Parkinson’s disease
PFF: preformed fibril
PrP: prion protein
SOD1: superoxide dismutase 1
TDP-43: transactive response DNA-binding protein 43.
